# ‘Time is of the essence’: upper-body aerobic exercise to improve cardiovascular health during inpatient rehabilitation within the first year following spinal cord injury – protocol for a randomised clinical trial

**DOI:** 10.1136/bmjopen-2024-089868

**Published:** 2025-04-30

**Authors:** Shane J T Balthazaar, Daniel D Hodgkiss, Shin-Yi Chiou, Samuel J E Lucas, Afroditi Stathi, Manish Kalla, Aheed E F Osman, Srinivasa C Budithi, Naveen Kumar, Joy Roy Chowdhury, Tom E Nightingale

**Affiliations:** 1 School of Sport, Exercise and Rehabilitation Sciences, University of Birmingham, Birmingham, UK; 2 University of Birmingham Institute of Cardiovascular Sciences, Birmingham, UK; 3 Department of Cardiology, University Hospitals Birmingham NHS Foundation Trust, Birmingham, UK; 4 International Collaboration On Repair Discoveries (ICORD), The University of British Columbia, Vancouver, British Columbia, Canada; 5 University of Birmingham Centre of Precision Rehabilitation for Spinal Pain, Birmingham, UK; 6 Centre for Human Brain Health, University of Birmingham, Birmingham, UK; 7 Department of Physiology, Anatomy and Genetics, University of Oxford, Oxford, UK; 8 Department of Cardiovascular Medicine, University of Oxford, Oxford, UK; 9 Robert Jones and Agnes Hunt Orthopaedic Hospital NHS Foundation Trust, Oswestry, UK; 10 Keele University Faculty of Medicine & Health Sciences, Keele, UK; 11 Edge Hill University, Ormskirk, UK

**Keywords:** Cardiovascular Disease, REHABILITATION MEDICINE, Neurological injury

## Abstract

**Introduction:**

Individuals with spinal cord injury (SCI) face heightened cardiovascular disease (CVD) risks. While general exercise guidelines are promoted for SCI individuals, when and how to incorporate exercise during the subacute phase post-SCI remains unclear. Consequently, early aerobic exercise to reduce CVD risks is not standard practice in subacute SCI care, potentially missing an opportunity for optimal cardiovascular rehabilitation, especially given observed reductions in cardiac structure and function within the first year post-SCI. Addressing this gap could improve long-term cardiovascular health and health-related quality of life (HRQOL) for individuals with SCI. Early intervention might prevent worsening cardiovascular function and establish beneficial exercise habits. However, few studies have evaluated the effectiveness of early exercise interventions in this population. This study aims to provide insight into the impact of moderate-intensity arm-crank exercise training (ACET) on cardiometabolic, HRQOL, functional and fitness parameters in individuals with subacute (<12 months postinjury) SCI.

**Methods and analysis:**

We will conduct a single-centre, two-group, single-blind randomised controlled trial with 42 participants who have sustained a cervical or thoracic SCI within the past year. The non-intervention group will receive hospital standard of care (control group) while the intervention group will receive hospital standard of care plus moderate-intensity ACET for 10 weeks in line with the SCI-specific exercise guidelines to improve cardiometabolic health. The primary outcome measure will be central arterial stiffness (carotid-to-femoral pulse wave velocity). Secondary outcomes include assessments of (1) blood biomarkers linked to CVD, (2) cardiac structure and function, (3) extracranial vasculature, (4) HRQOL, (5) cognitive function, (6) physical activity level, (7) cardiorespiratory fitness, (8) motor function and (9) feasibility outcomes. Assessments will occur at baseline (rehabilitation centre admission, −2 weeks), preintervention (0 weeks), postintervention (10 weeks) and follow-up (6 months after postintervention), for HRQOL outcomes only.

**Ethics and dissemination:**

Ethical approval was obtained from the Wales Research Ethics Committee (HREC 22/WA/0329). Outcome data will be presented at international conferences, patient advocacy groups, health professional networks and community health events. Findings will be published in peer-reviewed journals and widely disseminated through strategic channels to reach researchers, healthcare providers, patients and the public.

**Trial registration number:**

ISRCTN99941302.

**Strengths and limitations of this study**

- A longitudinal study design will allow us to look at changes in cardiovascular risk factors over time in the standard of care group (at a National Health Service (NHS) Spinal cord injury (SCI) rehabilitation centre) compared with the intervention group, who perform the population-specific exercise guidelines to try and mitigate declining cardiovascular health, thereby filling an important gap in understanding the benefits of early exercise interventions for people with SCI.
- The data collection in this trial encompasses measures of both traditional and non-traditional prognostic risk factors for cardiovascular disease.
- The outcome measures have demonstrated validity and reliability, specifically in individuals with SCI.
- Study recruitment and procedures will occur in one SCI rehabilitation centre to control for variation in the standard care provided to both groups; therefore, the findings may not be generalisable to all in-patient care settings in the UK.

## Background

 Spinal cord injury (SCI) is a life-altering event that can result in disruption to normal sensory, motor and/or autonomic functions,^1 2^ leading to a wide range of physiological and psychological consequences. Living with SCI can place a significant economic burden on patients, their families and the healthcare system, as it requires substantial healthcare resources.^3^ Recent improvements in technology, medical and rehabilitative care have led to individuals with SCI living significantly longer postinjury,^4^ with the odds of death during the first year postinjury being reduced by 67% for individuals injured between 1993 and 1998 relative to individuals injured between 1973 and 1977.^5^ Nevertheless, individuals with SCI are experiencing an accelerated trajectory of diseases and disorders that resemble those experienced with ageing alone.^6^

The dysregulation of the autonomic nervous system following SCI can play a role in the development of secondary conditions,^7^ contributing to the premature mortality in the SCI population being up to three times higher compared with the non-injured population.^8^ The primary cause for morbidity and mortality in individuals with SCI is the development of cardiovascular disease (CVD),^9^ which occurs earlier and affects over 60% of the SCI population.^10^ Early arterial remodelling represents a myriad of structural and functional changes of the vascular wall in response to sustaining an SCI, which has also been associated with haemodynamic changes and increased cardiovascular morbidity and mortality.^11^ Carotid-to-femoral pulse wave velocity (cfPWV), which is a measure of central artery stiffness, has been shown to be significantly elevated in individuals with chronic SCI compared with non-injured controls.^12^ This is of clinical relevance given that cfPWV has been directly linked with cardiovascular mortality in non-injured individuals.^13^

Reduced mobility as a result of both injury-related functional impairments and the need for medical stabilisation post-SCI can cause the cardiovascular system to undergo immediate alterations in its function as it adjusts to the new reduced metabolic demands.^14^ In as little as 6 weeks after injury, peripheral arteries in the paralysed lower limbs can undergo remodelling, resulting in reduced flow and diameter of the femoral artery.^15^ This remodelling is in conjunction with the diminished sympathetic tonic activity to the blood vessels below the lesion,^16^ which can result in profound hypotension. Moreover, individuals with higher-level injuries (ie, cervical and upper-thoracic injuries above the sixth thoracic level (≥T6)), often experience more pronounced hypotension due to potentially disrupted autonomic pathways.^17 18^ Beyond the acute pathological issues associated with blood pressure (BP) variability,^19^ chronic exposure to unstable BP has been shown to detrimentally impact cardiac and vascular structure and function,^6 20^ which can be measured via ultrasound. Assessing the autonomic regulation of the cardiovascular system non-invasively can be achieved using heart rate variability (HRV).^21^ Specifically, the frequency domain of HRV predicts cardiovascular dysfunction in individuals with SCI.^22^

SCI significantly contributes to obesity by altering fat-free mass and causing physical impairments such as paralysis or paresis, which restricts energy expenditure associated with movement, thereby increasing cardiometabolic risk.^23^ Such changes disrupt metabolic homeostasis,^24 25^ resulting in disorders such as insulin resistance and dyslipidaemia, which in turn increases the risk of developing type 2 diabetes mellitus and CVD in individuals with SCI.^26^ Individuals with both traumatic and non-traumatic SCI experience an elevated risk of CVD, highlighting the importance of routine CVD risk screening during rehabilitation for both groups.^27^

Reduced physical activity levels due to SCI-related immobility, coupled with decreased fitness and sympathetic nervous system dysfunction in individuals with injuries ≥T6, further limits the capacity to achieve higher energy expenditures through exercise.^28^ A Canadian-based study showed that individuals with SCI undergoing inpatient rehabilitation experience minimal cardiovascular stress, spending a median of only 5 mins/day performing activities at ≥40% heart rate reserve. This level of activity is not sufficient to obtain a cardiovascular training effect to optimise cardiovascular health, despite the participants self-reporting that they were doing a sufficient volume of exercise.^29^ Therefore, accurately assessing physical activity levels during the subacute period of SCI is also crucial for clinicians to tailor interventions to mitigate the aforementioned CVD risks in individuals living with SCI. However, accurately measuring physical activity levels in individuals with SCI is challenging due to the altered movement patterns and variations in metabolically active muscle mass.^30^ These challenges may be overcome by using an individually calibrated and validated multisensor device (as proposed in this trial) to provide valuable insight into the amount of physical activity achieved during inpatient SCI rehabilitation in the UK.^31^

Individuals with SCI often report a lower health-related quality of life (HRQOL) compared with non-injured individuals.^32^ Various factors influencing HRQOL following SCI include pain,^33 34^ fatigue^35^ and both cognitive^36^ and motor^37^ impairments. Common types of pain include musculoskeletal and neuropathic pain.^34^ Musculoskeletal pain, which can occur due to muscle spasm or the overuse of the arms and shoulders,^38^ can hinder independence and lead to reduced aerobic performance during activities of daily living and chronic postural deviations.^39^ Neuropathic pain is considered the most debilitating pain that is spontaneous in the majority of individuals with SCI and can manifest as increased pain with both noxious and innocuous stimulation.^40^ Pain is prevalent in 60%–80% of individuals with SCI, with one-third reporting severe pain.^40^ Chronic fatigue affects around 56% of individuals with SCI, which is almost double the rate observed in the non-injured population.^41^ Multiple factors contribute to cognitive impairments in individuals with SCI,^42^ including medications (eg, opioids such as pregabalin and gabapentin),^43 44^ traumatic brain injury, which occurs in almost 60% of individuals with high-level injury,^45^ and autonomic cardiovascular dysfunctions.^19 46^ Memory and learning impairments can be a challenge for the required development of new skills during rehabilitation.^47 48^ Therefore, enhancing HRQOL necessitates evaluating cognitive abilities, developing targeted rehabilitation strategies to mitigate pain and fatigue, while also improving motor function and ensuring that a substantial time period is devoted to promoting targeted physical activity outside of standard of care physical therapy.^49^,^52^

### Exercise interventions to improve cardiovascular health in individuals with subacute SCI

The cardiovascular stress during inpatient rehabilitation in the UK remains to be objectively quantified, as we have done previously to capture habitual free-living behaviours in community-dwelling individuals with SCI.^31^ Preclinical evidence indicates that early administration of exercise following experimental SCI leads to more pronounced cardiohaemodynamic benefits than delayed exercise administration.^53^ This is likely important in the clinical setting as literature indicates that individuals with SCI exhibit significant reductions in cardiac function within the first 6 months following injury.^54 55^ Arm-crank exercise training (ACET) is an inexpensive and widely used exercise modality for individuals with SCI^56^ that has been shown to improve cardiorespiratory fitness.^57^ Despite the amount of evidence supporting the benefits of ACET for cardiovascular health in chronic (>12 months) SCI,^57^,^59^ there is little understanding of whether the same effect can be expected in subacute SCI and whether an exercise intervention initiated in this stage postinjury mitigates declines in cardiac structure and function.^60^ A recent systematic review indicated very low levels of evidence for the benefits of exercise in the subacute phase following SCI,^58^ primarily due to the limited and poorly designed research studies available.

Given the known benefits of ACET for individuals with chronic SCI, this study aims to investigate the effects of exercise on cardiovascular health parameters in patients with subacute SCI. It is also essential to qualitatively assess the feasibility and implementation of ACET in the subacute rehabilitation setting. Understanding both patient and healthcare provider/staff perspectives on receiving and delivering an ACET intervention, respectively, is crucial for the development of effective rehabilitation tailored to individuals with SCI. While regular physical activity has been reported to have significant health benefits for individuals with SCI,^61^ it is equally as important to consider the potential overburdening of inpatients alongside their standard of care, as well as both the clinical and cost-effectiveness of promoting additional prescribed aerobic upper-body exercise in the rehabilitation pathway.^62^

### Trial objectives

We aim to determine whether the addition of ACET to standard of care treatment during inpatient rehabilitation within the subacute period following SCI has beneficial effects over the current standard of care (control group, CON) rehabilitation on arterial stiffness (measured via cfPWV) and secondary health outcomes: (1) blood biomarkers related to CVD, (2) cardiac structure and function, (3) extracranial vasculature, (4) HRQOL, (5) cognitive function, (6) physical activity level, (7) cardiorespiratory fitness, (8) motor function and (9) feasibility outcomes in individuals with subacute SCI. In comparison to CON, we hypothesise that the physical and metabolic stimuli of ACET will maintain cfPWV at a level that is comparable to normative age-matched values reported in the general population.^63^

## Methods and analysis

### Study design and setting

This is a single-centre, two parallel-group, randomised controlled trial of a 10-week ACET exercise intervention compared with standard of care inpatient rehabilitation with a 6-month follow-up assessment after discharge for HRQOL outcomes. This protocol is approved by the Integrated Research Application System (IRAS; 315098) and the design of the study is aligned with the consensus-based recommendations for designing, delivering, evaluating and reporting exercise-intervention research involving people with SCI.^64^ The Standard Protocol Items: Recommendations for Interventional Trials^65^ checklist can be found in the online supplemental material (Research Checklist). Participant eligibility to enter the study is within 12 months post-SCI to reflect a realistic representation of the SCI population undergoing rehabilitation in the UK. The site initiation visit was completed on 7 September 2023, with the first participant assessment in November 2023. Participant recruitment will stop in September 2025. The study will be conducted in accordance with ethical principles for studies involving human participants set out in the Declaration of Helsinki.

The study and all recruitment will be conducted at the Robert Jones and Agnes Hunt (RJAH) Orthopaedic Hospital. This SCI Rehabilitation Centre caters to a wide catchment area, serving a population of 10 million people that includes the West Midlands (where the University of Birmingham is situated). The Midland Centre for Spinal Injuries (MCSI) at RJAH offers a multidisciplinary approach to SCI treatment that focuses on maximising neurological recovery while minimising complications, ensuring a holistic approach to patient care. This includes a commitment to innovation to transform care, which aligns well with the objectives of the study to evaluate the efficacy of an ACET intervention.

### Recruitment and consent process

Participants will be identified by trained clinical staff on admission to the rehabilitation hospital, based on the eligibility criteria outlined in table 1. If patients express an interest in the study, a participant information sheet will be given to them along with the contact information of the research team should they have any questions. Interested and eligible participants will be approached by a study team member and asked to provide written informed consent (>24 hours after receiving the participant information sheet and receiving a full verbal explanation of the study). If participants are unable to provide a signature due to their injury, a witness will be present to sign confirming the prospective participant’s verbal consent.

**Table 1:** Participant inclusion and exclusion criteria

Inclusion criteria	Exclusion criteria
For patients: At least 18 years old Males and females Have had an SCI* (AIS A, B, C or D) between C5 and T12 levels within 12 months; injuries graded AIS D on consent must be using a wheelchair >75% of the time for mobility Are cleared by their medical team to begin standard of care rehabilitation. Can move their shoulders and arms voluntarily to operate the arm-crank ergometer. Compliance: understands and is willing, able and likely to comply with all study procedures and restrictions. Consent demonstrates an understanding of the study and willingness to participate, as evidenced by voluntary written informed consent. For healthcare providers/staff: Currently be a member of the medical team supporting subacute spinal cord injured patients at MCSI. Be at least 18 years old. Be involved in the clinical care/rehabilitation of patients (eg, clinician, occupational therapist or physical therapist)	Pregnant (women who become pregnant will be advised to notify clinical staff, and on notification, will be withdrawn from the trial) Under the age of 18 years. Have an SCI lower than the T12 neurological level. Have an SCI above the C5 neurological level, intubation, a tracheostomy in situ or requires mechanical ventilation. Medical complications or premorbidities from the injury that, in the opinion of the healthcare or research team, would restrict or prevent the participation in exercise rehabilitation, pose undue personal risk or introduce bias into the trial. Co-occurring traumatic brain injury or cognitive impairment that either impacts the ability to follow study instructions and/or provide informed consent. Unable to provide fully informed consent.

* Spinal Cord Injury Levels & Classification.

AIS, American Spinal Injury Association (ASIA) Impairment Scale; C, cervical; MCSI, Midland Centre for Spinal Injuries; SCI, spinal cord injury; T, thoracic.

### Eligibility criteria

#### Randomisation

**Figure 1:**
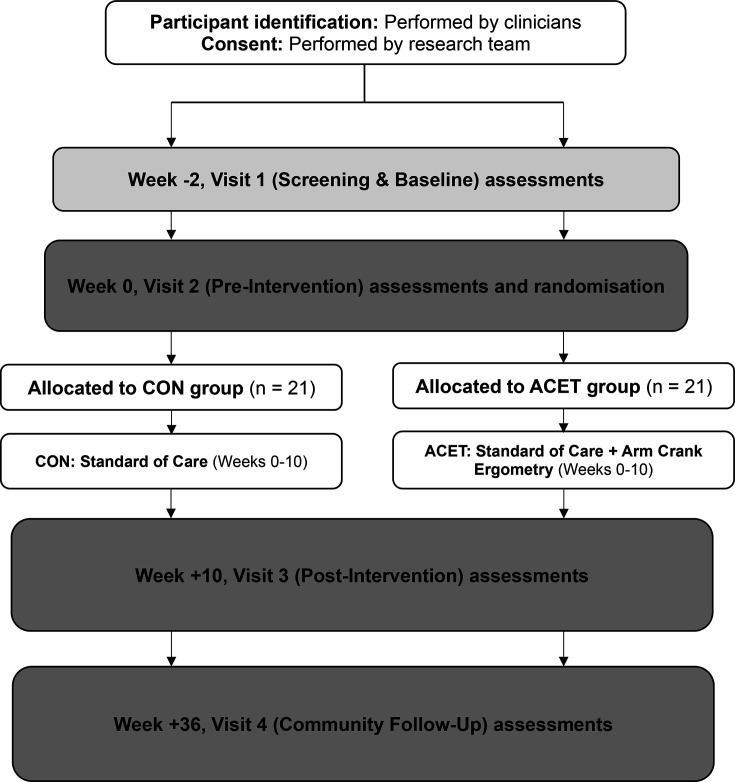
Trial flow chart. CON group, control group receiving standard of care in the rehabilitation setting; ACET group, intervention group undergoing arm crank ergometry training in addition to standard of care. ACET, arm-crank exercise training.

After preintervention assessments (A1 and A2), recruited participants will be randomly allocated into one of the two study groups using a computer-generated randomiser. Figure 1 provides an overview of the trial flow, illustrating the sequence of assessments, randomisation and subsequent testing. The A1 and A2 assessments are conducted prior to randomisation to gather initial data on participants to understand initial status and variability among participants. Conducting randomisation after these assessments minimises the risk of allocation bias, which could occur if knowledge of group assignment influenced initial measurements. Randomisation will be performed by an independent researcher (SJEL) not involved in the recruitment process to minimise selection bias. Block randomisation methods will be used to ensure balance in group sizes. In consultation with the Director of Statistics and Research Strategy at the Birmingham Clinical Trials Unit, it was determined that any imbalances of the participants recruited to the study would be down to chance. Each participant will be assigned a unique participant identification number and the corresponding trial group will be provided according to the list. Blinding is not achievable during data collection, therefore, neither participants nor data collectors will be blinded. However, to minimise bias, data analysts will be blinded to participant allocation.

### Patient and public involvement

Patients and community-dwelling individuals with SCI were not formally involved in the development of the trial (ie, formulating the research questions, design of the study or the selection of outcome measures). A patient with SCI reviewed the study protocol and provided a letter of support prior to the submission of the grant application. Further, participants will complete a standardised questionnaire regarding the acceptability and feasibility of the exercise intervention, described in detail in the Feasibility section below. Four open-ended questions will explore facilitators, barriers, benefits and suggestions for future exercise programmes at the subacute stage of SCI. Members of the study team are actively engaged with the wider SCI community and have experience with designing and conducting exercise interventions in this population.^66^,^68^

### Standard of care

After newly acquired SCI, patients are transferred from local trauma centres to one of the 11 specialised SCI centres in the UK, such as RJAH. Care at these centres adheres to established guidelines and best practices,^69 70^ providing patients with multidisciplinary support from a team of healthcare professionals. This team typically includes consultants (senior physicians), physiotherapists (to improve mobility and strength), occupational therapists (to improve independence in daily activities), dietitians and social workers.

The standard of care rehabilitation programme encompasses a broad range of services designed to address the physical, psychological and social needs of inpatients. Important aspects include the introduction to wheelchairs and adaptive aids, psychological support and education on self-care, such as skin integrity, bowel and bladder management and recognising potential complications. Patients have a minimum of 15 hours of rehabilitation per week, including appointments with a multidisciplinary team of clinicians and specialists, goal planning and case conferences.^70^ Additionally, the programme offers training for caregivers and family members, alongside tailored guidance on returning to work or exploring new opportunities, as appropriate.

Initially, postinjury and on admission, general strategies for preventing complications from spinal instability or neurological compromise are a priority. Skin integrity and bladder and bowel care are essential areas of focus for healthcare professionals, with no specific focus on addressing cardiovascular health per se. Currently, inpatient rehabilitation programmes aim to promote independence and facilitate optimal reintegration into the community.^71^

### Arm cycle ergometry training

ACET will be performed on an arm cycle ergometer (Monark 881E, Monark Exercise AB, Vansbro, Sweden) against individually determined levels of resistance. This initial exercise intensity will be prescribed as a power output that corresponds to ~55% and ~62% of each participant’s V̇O_2peak_ for individuals with paraplegia and tetraplegia, respectively, determined from their baseline graded cardiopulmonary exercise test (CPET). These population-specific values have been chosen as recent research indicates they more closely correspond to moderate-intensity exercise classifications than utilising non-disabled guidelines.^72^ This protocol can be successfully implemented in individuals with higher-level cervical SCI by using tensor bandages over Activehands grip aids to secure the participants’ hands to the ergometer.^73^ The height of the ergometer will also be positioned so that the crank apex will be at or lower than the height of the participants’ shoulders. Participants can self-select their arm-cycling cadence, but >70 revolutions per minute will be recommended.

**Figure 2:**
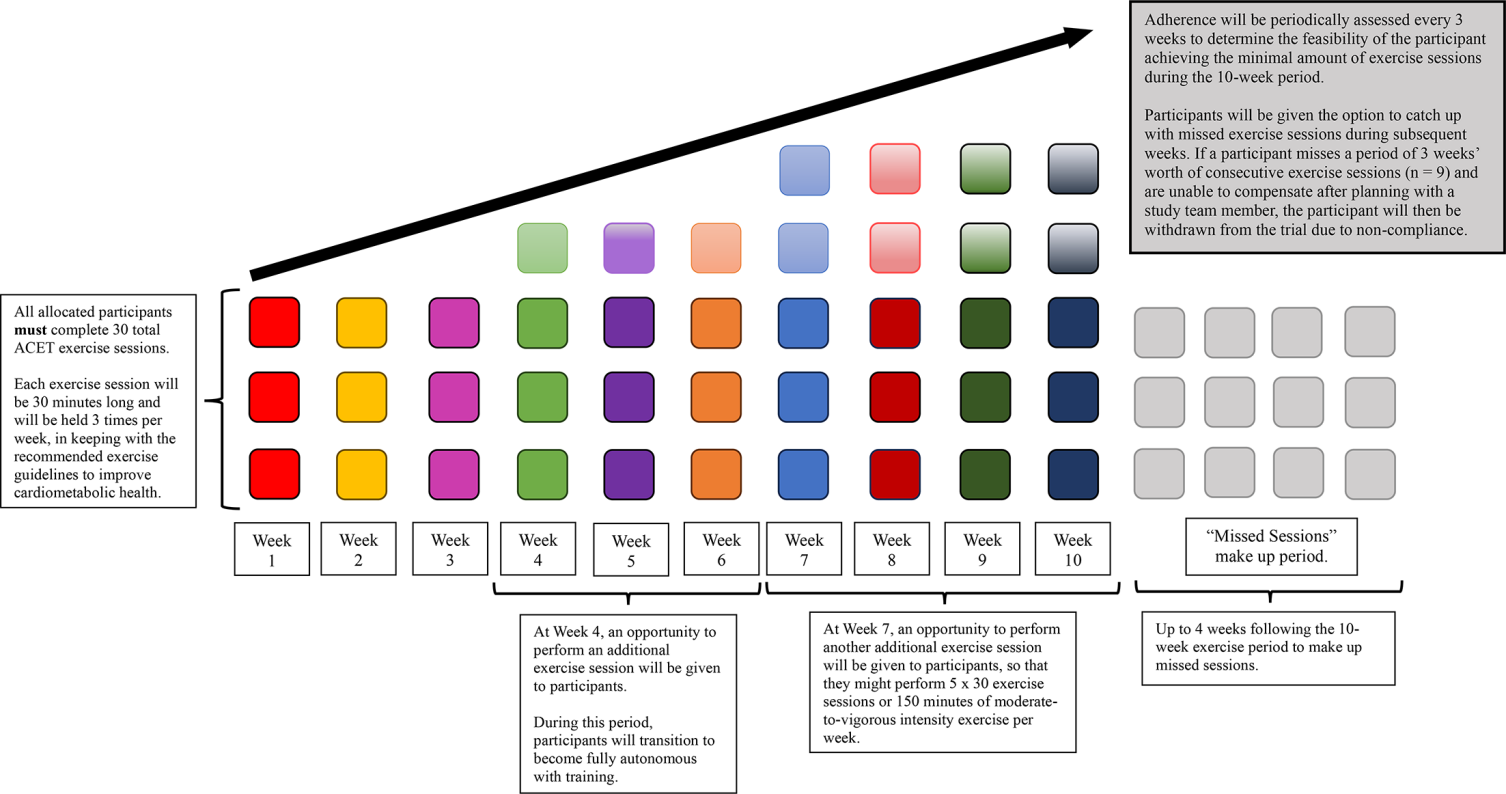
Adherence infographic. ACET, arm-crank exercise training.

Participants in the ACET group will initially perform 3×30 min per week of moderate-intensity arm-crank exercise, in keeping with SCI-specific exercise guidelines to improve cardiometabolic health.^74^ Participants will be allowed breaks in each exercise bout as required. By the end of the first week, participants should be able to cycle continuously for 30 min. At 4 and 7 weeks, the opportunity to perform an additional exercise session will be given to the participant, so that during weeks 7–10 of the intervention participants, could be performing 5×30 min per week of moderate-intensity ACET (figure 2). Such a gradual progression in exercise volume has been advocated in the Exercise and Sports Science Australia position statement on exercise and SCI,^75^ and ensures participants are achieving exercise guidelines promoted by reputable health authorities for the general population (eg, ~150 min of moderate-intensity exercise per week).^76^

For participants who choose not to perform the additional exercise sessions at 4 and 7 weeks, their decision will be respected and they will continue with the prescribed 3×30 min per week of ACET as outlined in the protocol, which is still in line with the minimal SCI-specific volume of exercise to improve cardiometabolic health.^74^ We will document the reasons for non-participation in the additional exercise sessions to inform future studies tailored to the SCI population. This approach allows us to identify the volume of aerobic exercise that is realistically achievable in the inpatient setting, and consequently, the individualised nature of the arm-crank exercise prescription enables us to determine what exercise training load is broadly achievable for patients with subacute SCI.

Should participants be discharged during the 10-week intervention period, they will receive an arm-crank ergometer (the same model during inpatient care) for continuing the intervention in their own homes for the remainder of the 10-week intervention. A member of the study team will visit the participant’s home to ensure proper setup of the device and supervise the first home-based session to ensure the correct exercise intensity is adhered to. Participants will wear a chest-worn HR monitor (Polar H10, Kempele, Finland), which will be paired to and stored on the cloud-based Polar Beat app (Polar, Kempele, Finland) via a commercial tablet (iPad, Apple, Cupertino, California, USA), while maintaining completed training logs for all arm-crank exercise sessions. Participants will receive a weekly phone call from a member of the study team to ensure compliance. This pragmatic approach has been used in another exercise trial performed with patients with SCI in the subacute setting,^77^ and considers the variability in injury rehabilitation duration (which depends on injury characteristics and medical complications) to ensure patients can complete the 10-week intervention. Exercise intensity progression will be regulated during the 10-week intervention for participants with paraplegia by adjusting the resistance during each session to achieve a target heart rate corresponding to moderate-intensity for each participant. Central (ie, overall effort and fatigue, accounting for the entire body’s sensation of exertion) and peripheral (ie, focused on localised sensations of effort and fatigue in the shoulders and arms) rating of perceived exertion (RPE) will be captured at the end of each exercise session, with RPE’s of 12–13 or 14–17 on a 6–20 Borg Scale corresponding to moderate or vigorous-intensity exercise, respectively.^72^ For participants with cervical injuries, if self-reported RPE is <12 the resistance for the subsequent exercise session will be increased to ensure an element of progressive overload is delivered, following consultation with the chief investigator (CI), who is an accredited clinical exercise physiologist (TEN; Academy for Healthcare Science Register ID: 61220). Perceptual regulation of exercise intensity using RPE has been shown to be a valid approach in the SCI population.^78^

During the first 4 weeks of the intervention, a research team member will adopt a flexible approach to scheduling supervised sessions, accommodating participant needs. The team member will assist with the physical setup for the arm-crank exercise, as well as provide verbal encouragement and guidance regarding exercise performance. In line with the consensus-based recommendations for exercise research in SCI,^64^ behaviour change techniques such as goal setting and planning with participants to set exercise goals during the session, using exercise logs to help participants track their progress, offering regular, constructive feedback during their exercise sessions and celebration of milestones (eg, completing a session with no breaks) will be implemented. BP will be monitored where individuals report symptoms of low or high BP (eg, light-headedness or headache) before or after exercise sessions during this initial 4-week period. At the start of week 5, participants will begin to transition to autonomous training with the goal to being fully autonomous by the end of week 6. During the transition period, the research team will provide only minimal guidance; for example, when participants struggle to remember proper setup or execution. This ensures that participants can independently perform exercise sessions over weekends when the weekly exercise session frequency increases towards the latter stages of the intervention. The transition from assisted exercise sessions to autonomous training reflects the principles of the self-determination theory,^79^ particularly in the context of autonomy and competence. Participants will be gradually given more control and responsibility over their exercise sessions to foster autonomy. As participants gain confidence and proficiency, the lessened reliance on external assistance promotes a sense of competence in their ability to exercise.

Adherence will be periodically assessed every 3 weeks to determine the feasibility of the participant achieving the minimal amount of exercise sessions during the 10-week period. This pragmatic approach accounts for the associated complexities of working with this population in the subacute setting that may result in exercise sessions being missed. Participants will be given the option to catch up with missed exercise sessions during subsequent weeks. If a participant misses a period of 3 weeks’ worth of consecutive exercise sessions (n=9) and is unable to compensate after review with a member of the study team, then they will be withdrawn from the trial due to non-compliance (figure 2).

### Assessments

**Figure 3:**
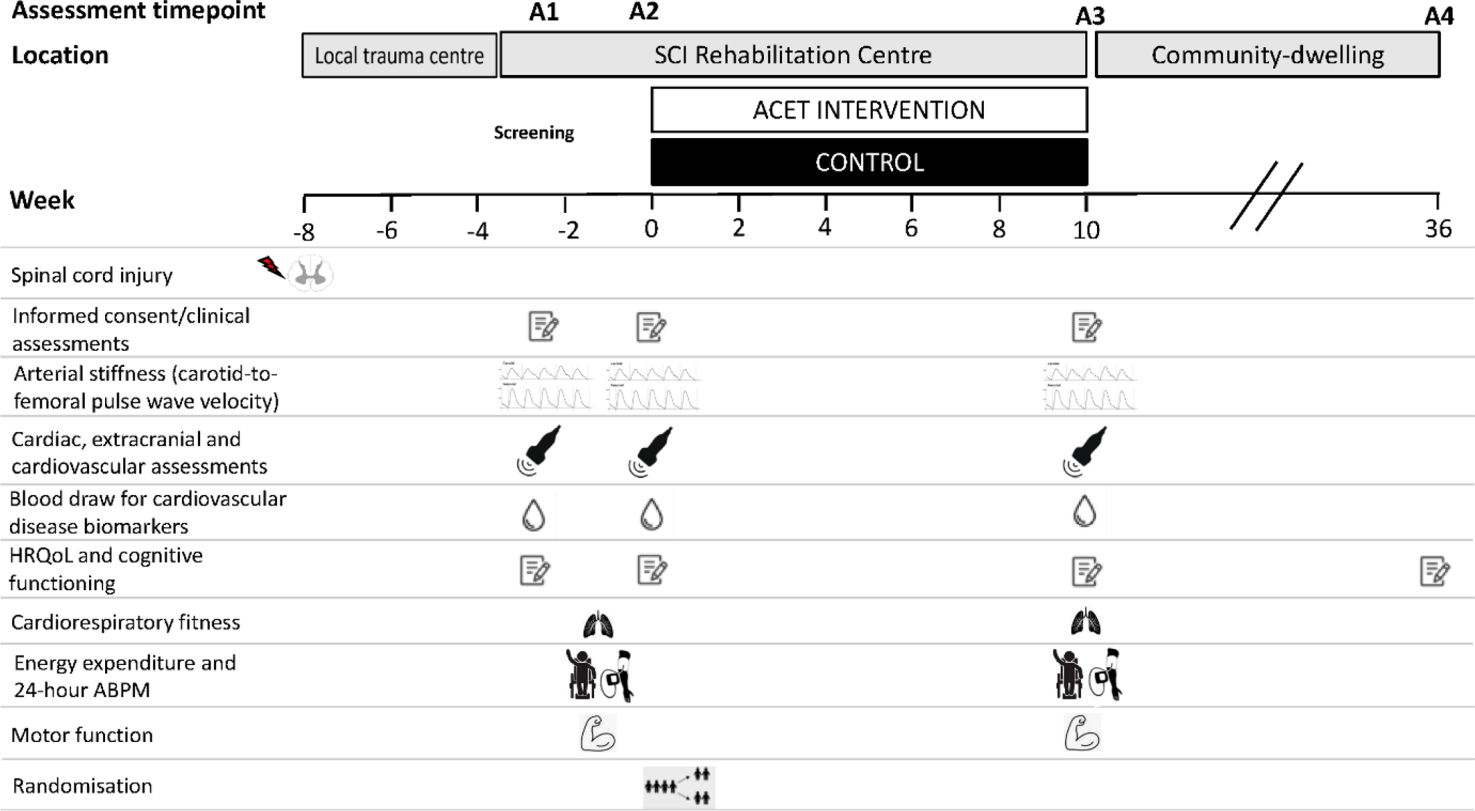
Study flow chart. Following participant identification and initial assessments (**A1**) at MCSI, assessments will be repeated after a period of standard care (which will primarily involve bed rest depending on the severity of injury) (**A2**). Participants thereby act as their own controls. At this point, participants will be randomised to receive either standard of care inpatient rehabilitation (CONTROL) or standard of care inpatient rehabilitation plus 30 min of ACET, 3–5 times per week (ACET INTERVENTION) for 10 weeks, with postassessments (**A3**). 6 months after discharge (~36 weeks) into the community, follow-up HRQOL assessments will be carried out (**A4**). ABPM, ambulatory blood pressure monitoring; ACET, arm-crank exercise training; HRQOL; health-related quality of life; SCI, spinal cord injury.

Following the provision of informed consent (see ‘Participant consent form’; online supplemental file 1), outcomes will be assessed at four time points: (A1) baseline (−>2 weeks), (A2) preintervention (0 weeks), (A3) following the ACET or standard care (postintervention) (10 weeks) and (A4) 6 months after discharge for HRQOL outcomes only (figure 3; table 1).

The A1 assessment serves as a time point for characterising key outcome measures on admission into inpatient rehabilitation, providing a baseline of cardiovascular health. Given the significant adjustments participants face following this life-changing injury, we have streamlined some of the secondary outcome measure assessments between A1 and A2 to minimise participant burden. Therefore, rehabilitation physical activity energy expenditure, 24-hour BP monitoring and motor function assessments will be conducted only once between A1 and A2, as outlined in table 2. The rationale for having both A1 and A2 assessments for the cardiovascular measures and HRQOL questionnaires is twofold: (1) to gauge any variability or changes in responses between the time close to admission (A1) and the adjustment to their new injury (A2; for example, following initial standard of care therapy sessions) and (2) to establish multiple baselines for these outcome measures, thereby allowing participants to serve as their own controls and capturing the inherent variability in these outcomes.

**Table 2:** Schedule of enrolment, interventions and assessments

Study procedures	Screen	Baseline(A1)	A1–A2	Preintervention/randomisation(A2)	Training sessions	Final week	Postintervention(A3)	Community follow-up(A4)*
Visit (V)	V1	V2	V2/V3	V3/V4	V4/V5–V45/V46	V46/V47	V47/V48	V48/V49
Eligibility								
Informed consent	X							
Medical history/demographics	X							
Changes in medication/health status				X			X	
Pregnancy screening	X							
International Standards for Neurological Classification	X						X	
Autonomic Dysfunction in Spinal Cord Injury	X						X	
Primary Outcome								
Carotid-Femoral Pulse Wave Velocity		X		X			X	
Secondary outcomes								
Blood sampling		X		X			X	
Health-Related Quality of Life Standard Questionnaires		X		X			X	X
Digit Span and Symbol Digit Modalities Test		X		X			X	
Wheelchair Users Shoulder Pain Index		X		X	X(weekly)		X	X
Cardiovascular Ultrasound		X		X			X	
Resting heart rate variability		X		X			X	
24-hour ambulatory blood pressure monitoring			X			X		
Rehabilitation energy expenditure (>3 days of monitoring) and free-living heart rate variability			X			X		
Motor function testing			X				X	
Arm cycle exercise familiarisation			X					
Cardiorespiratory fitness and heart rate recovery				X			X	
Continuous exercise heart rate and episodic (pre/post) Blood pressure monitoring					X(all sessions)			
Feasibility and Acceptability Questionnaire							X	
One-to-one Interview							X	

* For the A4 time point, only Health -Related Quality of Life Questionnaires and WUSPI will be completed.

Cardiorespiratory fitness assessments commence at A2 following a 20 min familiarisation session on the arm-crank ergometer, which will be performed by all participants between A1 and A2 (see table 2). The duration between A1 and A2 will be at least 2 weeks, although this timeframe is flexible based on the clinical needs of the patient. For example, prolonged bed rest may necessitate a longer interval before initiating standard of care rehabilitation due to factors such as fatigue or secondary complications such as pressure sores or urinary tract infections. At A4, occurring 6 months after A3 (ie, postintervention), we will remotely reassess HRQOL outcomes to determine whether there are differences between the ACET and CON groups.

A1, A2 and A3 assessments will be carried out within MCSI at the RJAH. A4 follow-up assessments will be conducted virtually. To ensure fidelity of assessments, the CI and research personnel held initial meetings for standardised training and logistics. Regular executive committee meetings consisting of the CI (TEN), site PI (JRC), site clinical lead (AEFO), postdoctoral research fellow (SJTB), research nurse, healthcare assistant and other MCSI staff will convene to discuss any data collection issues or safety issues that arise.

### Primary outcome measure: arterial stiffness

Measurements of cfPWV will be performed in accordance with international guidelines.^80^ Previously, cfPWV has been assessed in individuals with SCI.^20 81^ The Vicorder (Smart Medical, UK) system will be used with standard vascular cuffs, which has been shown to be a quick and highly reproducible technique for assessing cfPWV that is operator independent.^82^ Arterial pulse waveforms will be acquired by placing a 100 mm wide BP cuff around the upper thigh of the right leg to measure the femoral pulse and a 30 mm partial cuff around the neck at the level of the right carotid artery. The cuffs will each inflate to 60 mm Hg simultaneously to determine pulse transit time and cfPWV, with waveforms being recorded for at least 3 s while the participant is in the supine position. PWV is calculated as (0.8×*D*)÷Δ*t*, where *D* is the path length distance between the carotid and femoral sites and Δ*t* is the transit time.^80^ Path length is defined as the distance from the suprasternal notch to the top of the thigh cuff as indicated by the manufacturer. The use of cfPWV as a vascular health assessment tool was carefully considered in participants with anterior neck scars or cervical SCI with implanted metal hardware. Individuals with recent surgical scars or contraindications will be excluded from cfPWV assessments to ensure their safety.

### Secondary outcome measures

#### Blood CVD risk biomarkers

Moderate-intensity, upper-body exercise has previously been shown to decrease insulin resistance in individuals with chronic paraplegia.^83^ An 18.5 mL blood sample will be collected from the antecubital vein via a trained phlebotomist following an overnight fast (>10 hours). Serum and plasma samples will be dispensed into 1 mL aliquots and stored at −80°C for batch analysis at a later date using ELISA or an automated analyser (Randox RX Daytona). Biochemical outcomes relating to CVD risk will include triglycerides, total cholesterol, high-density lipoprotein cholesterol, low-density lipoprotein cholesterol, glucose, insulin and C- reactive protein.

#### Left ventricular structure and function

Cardiac structure and function has been shown to rapidly decline between 3 and 6 months during the subacute period of SCI.^54 55^ Furthermore, alterations to cardiac function and mechanics have been reported in individuals with chronic SCI following an exercise intervention.^84 85^ To measure cardiac indices, transthoracic echocardiography will be performed using a Vivid *iq* ultrasound system (General Electric Healthcare, Buckinghamshire, UK) in accordance with recommendations of the American Society for Echocardiography and European Association of Echocardiography.^86^ Images will be stored for offline analysis using specialised computer software (EchoPAC, General Electric Healthcare, Buckinghamshire, UK) by a single analyser. The average of three cardiac cycles will be used to determine left ventricular (LV) structural and functional indices. Indices of LV mechanics will be analysed using two-dimensional speckle-tracking software in accordance with recommended guidelines.^87^

#### Extracranial blood flow velocity and diameter

Functional adaptations to changes in blood flow in the common carotid artery (CCA) have been documented in individuals with SCI.^88^ To further investigate these adaptations earlier postinjury, blood velocity and vessel diameter of the right CCA, internal carotid artery (ICA), external carotid artery (ECA) and vertebral artery (VA) will be measured using a 12 MHz linear array probe (Terason uSmart 3300, Teratech, Burlington, Massachusetts, USA). The participant’s head will be turned slightly to the left side. Pulse-wave Doppler will measure peak blood velocity and B-mode will measure arterial diameter. The insonation angle will be set at 60° and will be unchanged throughout the collection of data.^89^ The ICA and ECA blood velocity and vessel diameter will be measured at least a distance of 1 cm from the carotid bulb to avoid turbulent flow patterns. VA blood velocity will be measured between the C4 and C6 vertebral level. Vessel location is based on an individual’s unique anatomical characteristics and will allow for reliable acquisition and repeatability on each assessment. Images will be stored for offline analysis using specialised software (Cardiovascular Suite 4, Quipu, Italy). Mean blood velocity will be measured from the velocity time integral.^90^ Mean shear rate will be calculated by four times peak velocity divided by vessel diameter.^91^ Subsequently, blood flow will be calculated using half the peak envelope velocity (ie, mean blood velocity) multiplied by the cross sectional area of the vessel.^92^

#### Resting heart rate variability

Resting HR will be measured continuously via five-lead ECG (Model 26T, ADInstruments, Colorado Springs, Colorado, USA). HRV will be measured in the time (eg, HR, SD of R-R intervals and the root mean square of successive R-R interval differences), frequency (eg, very-low-frequency, low-frequency, high-frequency and total power) and non-linear (eg, Poincaré plot analyses) domains. HRV represents a measure of the variation in time between heart beats as a time interval between the R waves of the QRS complexes,^93^ which are associated with overall health status due to increasing sympathetic tone.^94^ Before data are collected, each participant will rest in a supine position for at least 5 min in a quiet room. Resting HR will be recorded with unpaced, spontaneous breathing. Subsequently, in response to auditory cues for inspiration and expiration (ie, resonant breathing^95^), participants will record at least 5 min of data while they pace their breathing at a frequency of 15 breaths per min (0.25 Hz).^96^ Different breathing patterns have been shown to influence HRV metrics and therefore cardio–vagal balance.^97^

#### Health-related quality of life

Shoulder pain will be assessed at A1–A4 and weekly between A2 and A3 using the Wheelchair User’s Shoulder Pain Index (WUSPI)^39^ for individuals in the ACET group. The performance-corrected WUSPI score (PC-WUSPI) will be used as it accommodates participants who are unable to undertake certain functions. This correction multiplies the average response for all items by the number of questions attempted, with higher values indicating a greater degree of perceived shoulder pain. Bodily pain severity and interference will be assessed using the two-item Short Form-36 (SF-36) Pain subscale, capturing the past 4 weeks. Item scores will be combined into a single composite score (0–100 scale); lower scores indicate greater pain. Pain derived from the SF-36 subscale is commonly used in SCI clinical settings.^98^ Furthermore, experts working in the field have recommended this measure as a minimum to quantify pain in clinical trials,^99^ and it has shown acceptable reliability and validity in the SCI population.^100^ Fatigue^101^ will be measured via the Fatigue Severity Scale.

#### Cognitive function

A shortened neuropsychological test battery will be used that includes the Digit Span and Symbol Digit Modality Test (SDMT) to give a global indication of cognitive function. These tests will be delivered orally to account for motor function difficulties in individuals with higher-level SCI. Participants will verbally respond to the test stimuli with the same, trained assessor noting down their answers accordingly. The forward digit span measures attention and short-term memory, whereas the backward digit span task measures visuospatial integration and working memory. The SDMT measures complex visual tracking and working memory. Both of these cognitive tests have previously demonstrated good-to-excellent reliability when used in the SCI population.^102^ Previous work on cognitive health following SCI showed that up to 64% of injured individuals were cognitively impaired,^36^ with a 13-fold increased risk of cognitive impairment compared with non-injured individuals.^41^ Experimental SCI has been associated with structural alterations and endothelial dysfunction in cerebral arteries that possibly contribute to reduced cerebral blood flow and vascular cognitive impairment.^103^ While decentralised cardiovascular autonomic control has been implicated in cognitive and cerebrovascular deficits post-SCI,^104^,^106^ subclinical arterial stiffness has also been speculated to accelerate age-related cognitive decline.^46^

#### BP monitoring over a 24-hour period

Periodic (daytime: every 15 min; night-time: every hour) brachial BP measurements will be recorded using an ambulatory BP monitor (IEM Mobil-O-Graph). 24-hour ambulatory BP monitoring (ABPM) will be used to capture BP variability,^107^ plus will allow for the determination of nocturnal dip (considered a prognostic indicator for CVD^108^). As a diminished autonomic control over the cardiovascular system can cause profound BP instability, particularly in individuals with higher neurological levels of SCI, the use of ABPM allows for insight into circadian BP profiles and can provide prognostic values for cardiovascular morbidity and mortality in the SCI population.^109^ Furthermore, increased BP variability is a significant and independent predictor of mortality and cardiovascular events in the general population,^110^ and an increased frequency of hypotensive events over 24 hours has been associated with arterial stiffening^20^ and impaired cerebrovascular reactivity^106^ in individuals with SCI.

#### Rehabilitation energy expenditure and free-living heart rate variability

Participants will wear an individually calibrated multisensor device (Actiheart, Cambridge Neurotechnology, Papworth Everard, UK) for at least 4 days, which has been shown to be a sufficient duration to measure energy expenditure components in individuals with SCI.^31^ The Actiheart, which is worn on the chest and incorporates tri-axial accelerometry and HR, has been demonstrated to accurately and precisely estimate physical activity energy expenditure in wheelchair users.^111^ This device provides information on the number of minutes spent performing sedentary, light, moderate and vigorous-intensity activities.^112^ The Actiheart also has the capability of measuring HRV in different domains (as described above), with the advantage of the measures being taken over a 4-day period to provide insight on the integrity and responsiveness of the autonomic pathways in response to external stimuli.^17^

#### Cardiorespiratory fitness

Peak oxygen uptake (V̇O_2peak_) and peak power output (PO_peak_) will be determined using a graded CPET performed on an arm-crank ergometer until volitional exhaustion. Alternatively, to address the challenges of peak aerobic fitness testing in participants clinically determined to not be able to complete a maximal test in the opinion of the CI (a certified exercise physiologist), the Six-Minute Arm Test (6MAT) may be used. The 6MAT, which has been shown to reliably assess aerobic capacity in individuals with SCI, will be used to predict V̇O_2peak_ using an established regression equation.^113^ This approach will accommodate those unable to perform maximal exertion tests. Expired gases will be collected using a calibrated, portable metabolic cart (COSMED K5, Italy). BP will be measured before and after exercise. HR will also be measured throughout (rest, exercise and following 5 min of recovery). HR recovery (HRR) data will be used to determine vagal reactivation (fast phase) and sympathetic withdrawal (slow phase). Reduced HRR has been linked with increased cardiovascular morbidity and all-cause mortality.^114^ At least 3 days prior to performing the A2 CPET, participants in both groups will perform a familiarisation arm-crank exercise session at a self-prescribed intensity for 20 min. Therefore, participants will either perform a maximal exertion test or 6MAT for assessment of their cardiorespiratory fitness at the A1-A2 and A3 time points, which will not impact their randomisation to either arm of the trial.

#### Motor function

Prior work has indicated improved trunk control after upper-body exercise interventions, such as ACET.^68 115 116^ While the exact underlying mechanisms remain unknown, a possible explanation is to use the shared control mechanism between arm and trunk muscles which facilitate corticospinal excitability projecting to the trunk muscles during contractions of the arm muscles.^68 117^ To determine the effect of ACET on trunk motor function in subacute SCI, static and dynamic sitting balance for trunk stability will be evaluated using the Function in Sitting Test (FIST)-SCI.^118^ Individuals with SCI have ranked trunk stability as the third highest priority to improve life quality^119^ and this has been linked to the ability to perform functional activities after SCI.^118 120 121^ Muscle strength will be quantified by a hand-held dynamometer (Jamar Plus+, Performance Health, Warrenville, Illinois, USA) and reported in kilograms for participants with paraplegia only. These outcomes will capture loss or recovery in motor function and any improvements in strength, which is related to functional ability to perform activities of daily living.^122^

#### Feasibility outcomes

To obtain information about the usability and satisfaction with ACET in the subacute inpatient rehabilitation setting, participants will complete the Participant Evaluation of Feasibility and Acceptability Questionnaire,^123^ which consists of six items arranged on a Likert scale ranging from 1 to 5 and has been used previously for exercise interventions in individuals with SCI.^124^ Four open-ended questions have been developed and added to the instrument to identify facilitators for exercise; challenges/barriers to exercise; benefits experienced from participating in the ACET intervention; and suggestions for strategies to support individuals with subacute SCI to engage in ACET.

Framework analysis will be used to analyse the responses to the four open-ended questions using the NVivo qualitative research software (NVivo 12) to organise the data. A predetermined framework consisting of the four question topics (facilitators, barriers, benefits and recommendations) will be employed as an initial coding frame.^125 126^ This questionnaire will only be completed at A3 (see figure 1).

#### Longitudinal qualitative data collection

Participants in the ACET group will be invited to attend a one-to-one semistructured interview at completion of the intervention (A3) to discuss their views on the intervention received and the impact of the intervention on their recovery. The discussion will cover the study protocol, effects of the intervention on their health in the short term and the feasibility of administering the intervention in a hospital setting. The findings of the analysis of the four open questions will be used to probe further during the interviews.

In-person focus groups will also be performed with staff (clinicians, occupational therapists and physical therapists) involved in clinical care/rehabilitation of patients at RJAH on study completion to ascertain the acceptability of the intervention, trial design and outcome measures, as well as the staff’s experiences during the trial. Prior to taking part in the focus group, RJAH staff will complete a screening survey, requesting descriptive characteristics such as age, sex, qualifications, job role and level of experience. A trained moderator will run the interviews/focus groups and data will be audio recorded via a secure password protected digital recording device. These interviews/focus groups will address the aforementioned themes via a semistructured interview schedule.^127^ The recorded discussions will subsequently be transcribed verbatim and analysed employing the same framework analysis protocol used in the analysis of the open-ended questions as an initial coding frame.^126^

### Data management and confidentiality

Consent forms are not anonymised and will, therefore, be kept in a secured and locked place at the clinical research facility with access only to the research team. Questionnaire responses and results sheets will be stored as paper (A1 – A3) or electronic copies (A4). Access to participant data will be granted to the research team only. Hard copies of these documents will be secured in a locked cabinet within clinical research facilities at the MCSI. All protocol contributors named in this document will have access to the final trial dataset.

Electronic data will be anonymised and stored on a password-protected university computer. A laboratory laptop computer (owned by the University of Birmingham) will only be used to conduct non-identifiable analysis of results. The data from blood samples (for analysis of CVD risk biomarkers) will be analysed by the research team in the School of Sport, Exercise and Rehabilitation Sciences. All samples will be stored with the participant code and time point, with a sample inventory created to allow for easy sample location at a given time. Deidentified study data, coded with a unique study ID assigned to each participant, will be kept at the University of Birmingham. The study-specific folder can only be accessed by the research team. Hard copies of trial data will be transported and stored in a locked cupboard at the University of Birmingham. Study data will be kept for 10 years in line with the University of Birmingham research data policy. Participants’ samples will be stored in accordance with the Human Tissue Act, 2004. Any spare stored samples may be used in the future by either the research team or their collaborators to further explore the changes in cardiometabolic health following SCI and how this might be mitigated with aerobic exercise in the subacute setting. This is only applicable to participants who have provided specific consent for this. Where this is the case, further ethical approval will be sought for additional analyses based on the General Data Protection Regulation (GDPR). Specifically, a Data Use Agreement will be established to ensure the purpose of data use, scope of data access, data security measures and any data sharing restrictions. Anonymised data will be made accessible in a public repository on request while adhering to GDPR requirements, such as providing privacy notices.

### Statistical analysis

The Shapiro-Wilk test will be used to test the distribution of the continuous variables for demographic and outcome measures. Continuous variables will be reported as mean±SD for normally distributed data or median with IQRs for non-normally distributed data; categorical variables will be reported as frequency. Specific injury characteristics will be considered for subanalysis (ie, injury level, injury severity). A two-way repeated measures analysis of variance (ANOVA) will be applied to examine the main effects of the group/time and group–time interaction. Post hoc analyses will be conducted where significant group-time interaction effects are identified. Significance level will be set at 0.05 for all statistical tests. Standardised effect sizes (Cohen *d*) will also be calculated and interpreted based on the following criteria: small (*d*=0.2), medium (*d*=0.5) and large (*d*=0.8) effects.^128^

### Power calculation and sample size

We aim to recruit 42 participants for this study. This sample size was based on the effect size drawn from the CHOICES multicentre RCT.^129^ Our a priori power calculation indicated that a sample size of 32 individuals with SCI, 16 in each group, is sufficient to detect a between-group interaction effect for cfPWV, using a two-way repeated measures ANOVA with 95% power and a 5% level of significance to detect exercise group differences of ≥1 m/s. Our target effect size of 1 m/s aligns with reported exercise intervention effects in high-risk populations and represents a clinically meaningful improvement, as each 1 m/s increase in cfPWV corresponds to a 15% increased risk of cardiovascular mortality.^130 131^ A recent study found that ~16% of participants who were randomly assigned to a 5-week ACET intervention in the subacute setting discontinued the intervention.^132^ To account for the longer intervention duration in this trial, a more conservative (30%) dropout rate was estimated. Hence, 42 participants in total will be enrolled in the study.

### Safety

The trial has been designed to reduce participant risk and burden as much as possible, with strict adherence to best practice guidance for all methods. Any adverse events (AEs) reported will be documented with information pertaining to their severity and anatomical location and the CI will classify the AE according to internationally recognised definitions^64^ and promptly report these to the appropriate regulatory bodies and ethics committees as required. Any serious AEs (SAEs) will be immediately reported to the CI, who will assess the relatedness to the intervention. Reports of related and unexpected SAEs will be submitted to the Research Ethics Committee and sponsor (University of Birmingham).

### Ethics and dissemination

This study was approved by the Wales Research Ethics Committee 2 (REC reference number 22/WA/0329; approved on 9 November 2022) and registered on the International Standard Randomised Controlled Trial Number registry on 5 May 2023 (ISRCTN99941302). For substantial and non-substantial amendments, the sponsor (University of Birmingham) will sign off on any alterations before submission to IRAS. On completion, the CI will notify the relevant parties and study site of any alterations to the study. The data arising from this trial will be submitted for publication and presented at international conferences (eg, International Spinal Cord Society, American Spinal Injury Association, American College of Sports Medicine). People will be notified of the outcomes of the trial via lay press releases or blogs written for UK SCI charities and support groups. Extensive, individualised feedback will be provided to each study participant following the 6-month discharge period.

## Discussion

Individuals living with chronic SCI in the UK report being satisfied or very satisfied with their life as a whole and various aspects of their life, but often report challenges related to occupation and physical independence,^133^ highlighting the need to investigate alternative rehabilitation strategies to promote functional independence after injury. Given the high prevalence of detrimental health consequences associated with SCI, the implementation of early and effective therapeutic interventions targeting cardiovascular health is crucial.^54^

Current management of SCI mainly focuses on functional rehabilitation and does not include any formal prescription of an aerobic exercise programme to minimise cardiovascular dysfunctions and CVD risk.^134^ However, initiating an exercise intervention early in the subacute phase of inpatient rehabilitation after SCI (ie, within 12 months) could serve as a preventive measure to mitigate the increased CVD risk associated with SCI.^135^ The outcomes of this study have the potential to provide evidence of whether implementing early cardiovascular-focused exercise interventions in the subacute stage of injury could be a safe and effective approach to prevent secondary complications associated with SCI and enhance independence in performing activities of daily living for individuals with SCI.

The early rehabilitation environment represents the ideal time and location to embed healthy lifestyle behaviours, including upper-body exercise conditioning.^77^ Previous research has demonstrated that moderate-intensity exercise interventions have induced favourable adaptations in untrained individuals with chronic SCI, such as improved functional capacity (ie, cardiorespiratory fitness and peak power output) and a range of HRQOL measures.^66 136 137^ Moreover, these types of interventions may result in reduced healthcare expenses after discharge from inpatient rehabilitation, which can be an average lifetime cost of £1.12 million per SCI case in the UK.^138^ Furthermore, this moderate-intensity exercise intervention has broader implications for addressing mobility issues and mitigating CVD risk in various inpatient groups.

One of the strengths of this study is that the intervention programme was constructed based on existing evidence and exercise guidelines for individuals living with SCI.^74^ By addressing limitations observed in previous studies during subacute rehabilitation, our study aims to generate high-quality evidence to answer whether early aerobic exercise rehabilitation benefits cardiovascular health outcomes. In contrast to some exercise intervention studies that overlook participant experiences, our trial will assess feasibility and acceptability through postintervention interviews with participants to inform future clinical rehabilitation interventions during the subacute phase of SCI. Such qualitative data will provide contextual information on the views of the participants and staff working at RJAH. Lastly, the double-baseline assessments for some of our outcome measures allow us to better understand the inherent variability of these outcomes in the inpatient setting. However, we acknowledge the limitations of this study. Blinding of participants and the study investigator cannot be performed due to the awareness of the randomised intervention. Nevertheless, data analysts will be kept blind to group allocation, which may decrease the risk of detection bias during the study’s implementation. There is also the possibility that participants in the CON might independently engage in ACET or other physical activity behaviours, which could potentially confound the results. Lastly, study recruitment and procedures will be conducted solely in one SCI Centre, potentially limiting the generalisability of the findings to other centres in the UK. Despite these limitations, the trial will contribute valuable insights to advance our understanding of mitigating CVD risk in newly injured individuals with SCI.

## Supplementary material

10.1136/bmjopen-2024-089868 online supplemental file 1
