# Supplementary material for: ‘Time is of the essence’: upper-body aerobic exercise to improve cardiovascular health during inpatient rehabilitation within the first year following spinal cord injury – protocol for a randomised clinical trial
Source: BMJ Open. 2025 Apr 30;15(4):e089868. doi: 10.1136/bmjopen-2024-089868 (PMC12049926; doi:10.1136/bmjopen-2024-089868)
Supplement: Supplementary file 2 [file bmjopen-2024-089868.reviewer_comments.pdf]

PEER REVIEW HISTORY

BMJ Open publishes all reviews undertaken for accepted manuscripts. Reviewers are asked to complete a checklist review form and are provided with free text boxes to elaborate on their assessment. These free text comments are reproduced below.

ARTICLE DETAILS

Title (Provisional)

"Time is of the essence" - Upper-body aerobic exercise to improve cardiovascular health during in-patient rehabilitation within the first year following spinal cord injury: Protocol for a randomised clinical trial

Authors

Balthazaar, Shane J.T.; Hodgkiss, Daniel D; Chiou, Shin-Yi; Lucas, Samuel JE; Stathi, Afroditi; Kalla, Manish; Osman, Aheed E.F.; Budithi, Srinivasa C.; Kumar, Naveen; Chowdhury, Joy Roy; Nightingale, Thomas

---

VERSION 1 - REVIEW

**Reviewer**	**1**
**Name**	**Keessen, Paul**
**Affiliation**	**Amsterdam University of Applied Sciences, Faculty of Health**
**Date**	**28-Oct-2024**
**COI**	**None**

I want to congratulate the authors with the protocol of this important study. This study is of great importance and will give valuable information. There are some points need to be addressed.

Introduction

The authors chose to focus solely on exercise training, which is very important. However, in addition to exercise training, a dietary intervention might help with better recovery. Did the authors consider adding a dietary intervention to this intervention?

Methods:

The authors provide a very detailed description of the intervention. However, a thorough explanation of standard care is lacking. Please elaborate.

A very important limitation to this study is the lack of blinding. This does raise an ethical question. Early exercise training might lead to better outcomes for patients with SCI. How do the authors deal with patients in the control group that want to participate in exercise training?

**Reviewer**	**2**
**Name**	**Sisto, Sue Ann**
**Affiliation**	**UB - South Campus, Rs**
**Date**	**19-Nov-2024**
**COI**	**None**

1. A shorter FU would be beneficial (3 mons) for secondary outcomes. 6 mos. is too long for HRQOL as so many life factors could influence this outcome.

2. Acute SCI is typically identified as up to 6 most, not 12 mos.

3. Many usual care (CONTROLS) may engage in ACET as part of their care and there is no mention of this. It is not possible to restrict usual care but it is not addressed at all.

4. There is a question of whether AIS A C5 could complete the test.

5. It is not clear why a HIIT protocol was not chosen but cited a HIIT paper (citation 74)

6. It is unclear for Cx injuries whether the RPE is for the ACET group of <12 that the resistance would be increased, is realistic,

7. While the secondary outcomes are reduced pre-ACET, they are not post-ACET and this might lead to loss of data.

8. Page 11, it is unclear why a longer interval before starting the study for Controls would be any different than the ACET assigned group.

9. Page 13, there is no reference for the ctPWV for use in SCI and this is the same of Left ventricular structure and function section.

10 Page 13, there is a reference for cognitive decline in SCI cognitive decline and one the reference on arterial stiffness in aging but no citation on SCI arterial stiffness and cognitive decline.

11. There is not DSMB mentioned wish would be essential in this clinical trial.

Page 14, there is no mention of risk of using the cfPWV on anterior neck scars and metal hardware for Cx injuries.

Page 15, a citation is needed on the prognostic value of 24 hour BP monitoring, not just a methodology citation

Page 17, mention of using spare samples for other CVD health question does require permission by participants.

Page 17, I could not find the ref. on CHOICES RCT (#118).

VERSION 1 - AUTHOR RESPONSE

<u>Reviewer #1:</u>

Dr. Paul Keessen, *Amsterdam University of Applied Sciences, Amsterdam UMC Locatie AMC*

I want to congratulate the authors with the protocol of this important study. This study is of great importance and will give valuable information. There are some points need to be addressed.

<u>Response:</u> Thank you for taking the time to review our manuscript. We are pleased that the reviewer agrees with the importance of this topic and the valuable information this trial will provide.

Introduction

The authors chose to focus solely on exercise training, which is very important. However, in addition to exercise training, a dietary intervention might help with better recovery. Did the authors consider adding a dietary intervention to this intervention?

<u>Response:</u> While we agree that nutrition plays an important role in the SCI population, our primary aim is to look at the effects of incorporating the upper body exercise training to the current standard of care model in the UK inpatient rehabilitation setting. While adding a dietary intervention would be valuable, this could introduce a confounding variable that could make it challenging to draw conclusions on the efficacy of exercise alone. Furthermore, at this stage post-injury, including a dietary intervention would require additional participant compliance and monitoring, which could increase participant burden and potentially reduce adherence to the exercise protocol.

Methods:

The authors provide a very detailed description of the intervention. However, a thorough explanation of standard care is lacking. Please elaborate.

<u>Response:</u> Thank you for your comment. We agree that a clearer description of the standard of care is important for interpreting study results and contextualising the importance of our study. In our revised manuscript (Page 9), under the <u>Standard of care</u> section, we have detailed that the standard of care for the participants in the Control group was consistent with clinical guidelines and best practices at the clinical site (Midland Centre for Spinal Injuries based at the Robert Jones and Agnes Hunt Orthopaedic Hospital) and typically included access to doctors, physiotherapists (to improve mobility and strength), occupational therapists (to improve independence in daily activities), dietitians and social workers. The rehabilitation program also includes introduction to wheelchairs and other aids, psychological support, education on skin care, bowel and bladder management, and recognising complications. Finally training for caregivers and family members is provided alongside guidance on returning to work or exploring new opportunities as appropriate (National Institute for Clinical Guidelines, 2016, https://www.nice.org.uk/guidance/ng41/resources/spinal-injury-assessment-and-initial-management-pdf-1837447790533; British Society of Physical & Rehabilitation Medicine, 2022, https://www.bsprm.org.uk/wp-content/uploads/2022/10/SCI-Rehab-standards.pdf). Thus, the current provision lacks formalised aerobic exercise training as standard practice. The physical demands of the current standard of care provision will be captured for participants using validated wearable devices (Actiheart^{TM}). We have directed the reader to two key references summarising the standards for specialist rehabilitation of SCI in the UK and specifically at the NHS study site. The edited text is now substantially more detailed than the two sentences included on standard of care rehabilitation in a previous sub-acute SCI exercise intervention protocol paper published in BMJ Open (Reference #77).

A very important limitation to this study is the lack of blinding. This does raise an ethical

question. Early exercise training might lead to better outcomes for patients with SCI. How do the authors deal with patients in the control group that want to participate in exercise training?
Response: Thank you for this important consideration regarding the lack of participant blinding and potential impact on participants in the control group. We agree that this is an important limitation of the study design, and addressing the needs and preferences of participants in the control group ensures ethical conduct in the study. While blinding cannot be achieved due to the exercise nature of the intervention, this limitation has been acknowledged and discussed in the manuscript. Regarding the participants that wish to participate in exercise training, they are not restricted from engaging in independent exercise or other rehabilitation activities (although this is not actively prescribed by staff, see above response to standard of care comment). Any activity that participants in the control group might perform will be quantified via the Actiheart™ device to monitor their energy expenditure for comparative purposes.

Reviewer #2:

Dr. Sue Ann Sisto, *University of Buffalo – South Campus*

1. A shorter FU would be beneficial (3 mons) for secondary outcomes. 6 mos. is too long for HRQOL as so many life factors could influence this outcome.

Response: We appreciate the observation regarding the follow up duration and how it might influence the study outcomes. We agree that lifestyle factors could be an influence on these outcomes, however, as a goal of this study is to foster autonomy for participants allocated to the ACET group and to assess if implementing a structured exercise program early on into their care pathway would have benefits as they integrate themselves back into the community. Therefore, we selected a six-month timepoint to capture sustained changes in HRQOL. As the participants are discharged into the community, a shorter follow-up period may risk missing the longer-term trends or stabilised outcomes, instead reflecting issues such has not having adequate care provisions or accessible adaptations to their living environments.

2. Acute SCI is typically identified as up to 6 most, not 12 mos.

Response: Thank you for your comment. We agree with this point and have now labelled the term for this period of SCI to "sub-acute" throughout our manuscript.

3. Many usual care (CONTROLS) may engage in ACET as part of their care and there is no mention of this. It is not possible to restrict usual care but it is not addressed at all.

Response: Thank you for highlighting the potential for the participants in the control group to engage in ACET as part of their routine care. We acknowledge that participants in the control group might independently engage in ACET or other physical activity behaviours, which could potentially confound the results. While not ethical to restrict usual care in a real-world study setting, we are monitoring their energy expenditure periodically via the Actiheart™ device. Please see our above response to Reviewer 1's Comment #4 regarding this point.

4. There is a question of whether AIS A C5 could complete the test.

Response: Thank you for raising this important concern. The functional capabilities of individuals with cervical SCI can vary significantly depending on the level and completeness of injury. During baseline assessments, in consultation with the medical team, inpatients interested in participating in the study will be evaluated to determine their suitability for participation. As outlined in Inclusion Criteria #5 (Table 1 in our manuscript), participants must be able to voluntarily move their shoulders and arms to operate the arm-crank ergometer.

We acknowledge the limitations of the American Spinal Injury Association Impairment Scale (AIS) classification, which can include individuals with active wrist dorsiflexion (C6 key

myotome) and elbow extension (C7 key myotome) due to the zone of partial preservation. C5 AIS A participants may exhibit several myotomes and dermatomes below C5 with > grade 3/5 power, provided there is loss of function in the S4 and S5 regions (Deep Anal Pressure and Voluntary Anal Contraction). Furthermore, cardiovascular-related mortality is the third leading cause of death in individuals with SCI, with cervical SCI being the most common and therefore the most susceptible to early mortality (DeVivo MJ, Stover SL. 1995. Long-term survival and causes of death. In: SL Stover, JA DeLisa, and GG Whiteneck (Eds.), Spinal cord injury: Clinical outcomes from the model systems, Chapter 14). This highlights the importance of assessing cardiovascular fitness and addressing modifiable risk factors in this population.

5. It is not clear why a HIIT protocol was not chosen but cited a HIIT paper (citation 74)
Response: Thank you for your comment. While our study does not use HIIT exercise, the reasoning behind the citation of this paper (now Reference #77, found on Page 10 of the current version of our manuscript) was to highlight the pragmatic approach to deliver the trial if patients are discharged during the 10-week period and how they are followed up and monitored. This recently published protocol uses an identical approach, which validates our pragmatic delivery of an exercise intervention within a UK SCI inpatient rehabilitation setting.

6. It is unclear for Cx injuries whether the RPE is for the ACET group of <12 that the resistance would be increased, is realistic,
Response: Thank you for your observation regarding the use of RPE as a guide for adjusting the resistance during the exercise intervention for participants with cervical SCI. We acknowledge the challenges for such participants. An aim of this study is to explore the feasibility of administering exercise training within the sub-acute setting. In part, this includes exploring how the important component of progression can be implemented into the intervention based on the need to adjust workloads as participants improve fitness and perceive the exercise sessions to be easier. We do not expect to face prescription challenges using RPE, given the individualised nature of it as an outcome measure. Each participant will be informed to provide honest feedback of how hard they perceive each session to be and the resistance will only be increased should they report an RPE <12. Nonetheless, we will provide details of these observations in the future trial outcome manuscript to clarify how the protocol was applied in practice and whether adjustments were necessary (or difficult to prescribe) for participants with cervical injuries, for example.

7. While the secondary outcomes are reduced pre-ACET, they are not post-ACET and this might lead to loss of data.
Response: While we are not sure what has led the Reviewer to provide this comment, Table 2 outlines the schedule of enrolment, interventions and assessments. We wish to clarify that all secondary outcomes are performed at least once pre-ACET and once post-ACET. As highlighted under Assessments on Page 11, *"we have streamlined some of the secondary outcome measure assessments between A1 and A2 to minimise participant burden… rationale for having both A1 and A2 assessments for cardiovascular measures and HRQOL questionnaires is two-fold: 1) to gauge any variability or changes in responses… 2) allowing participants to serve as their own controls and capturing the inherent variability in these outcomes."*

8. Page 11, it is unclear why a longer interval before starting the study for Controls would be any different than the ACET assigned group.
Response: We apologise to the Reviewer for any confusion on Page 11. We clarify from Page 11 that *all* participants serve as their own controls between A1 and A2 before being allocated

and randomised to the ACET or CONTROL group. The duration between A1 and A2 is variable but will be at least two weeks for repeatability of cardiovascular measures and HRQOL questionnaires. However, as the Reviewer is aware of in this population, other complications may arise in which testing might not be feasible due to medical complications such as pressure sores or urinary tract infections which would impact the validity of the cardiovascular measures (e.g., blood pressure) and questionnaires (e.g., pain). Therefore, the timeframe is flexible between these A1 and A2 measurements.

9. Page 13, there is no reference for the ctPWV for use in SCI and this is the same of Left ventricular structure and function section.

Response: Thank you very much for your comment regarding a reference for cfPWV in the SCI population. cfPWV has been assessed in individuals with SCI across several publications (Hubli et al., 2014, DOI:10.1179/2045772314Y.0000000232; Currie et al., 2019, DOI:10.1038/s41393-019-0316). This information and accompanying references have now been added to the manuscript. Regarding left ventricular structure and function, our current References #54 and #55 discuss this assessment in individuals with SCI, but have now included references #84 (Matos-Souza *et al.*, 2011, DOI:10.1038/sc.2010.88) and #85 (Balthazaar *et al.*, 2024, DOI:10.46292/sci24-00002) to highlight notable changes following exercise in individuals with SCI.

10 Page 13, there is a reference for cognitive decline in SCI cognitive decline and one the reference on arterial stiffness in aging but no citation on SCI arterial stiffness and cognitive decline.

Response: Thank you for your comment regarding specific references to link SCI, arterial stiffness and cognitive decline. While the manuscript includes references addressing cognitive decline in SCI and the relationship between arterial stiffness and aging, as the Reviewer has pointed out, we recognise the need to include evidence to address arterial stiffness and its potential link to cognitive decline. We have now addressed this by citing preclinical evidence (Sachdeva *et al.*, 2020, DOI:10.1089/neu.2019.6913) and a relevant review (Wecht & Bauman, 2009, DOI:10.1179/2045772312Y.0000000056). Furthermore, we have extended this discussion to include evidence from the clinical SCI population. Specifically, we have revised the text under Cognitive function, which will now state, *"Experimental SCI has been associated with structural alterations and endothelial dysfunction in cerebral arteries that possibly contribute to reduced cerebral blood flow and vascular cognitive impairment (Sachedeva et al, 2020). While decentralised cardiovascular autonomic control has been implicated in cognitive and cerebrovascular deficits post-SCI (Ji et al., 2024), subclinical arterial stiffness has also been speculated to accelerate age-related cognitive decline (Wecht & Bauman, 2009)."*

Regarding the Reviewer's comment about the methodology section, we acknowledge that our initial integration of arterial stiffness, cerebral haemodynamics, extracranial blood flow and cognitive function may have lacked sufficient clarity. To address this, we have now modified the introductory sentence to the Extracranial blood flow subsection to specify our interest in the functional adaptations in the sub-acute period: *"Functional adaptations to changes in blood flow in the common carotid artery (CCA) have been documented in individuals with SCI (Schmidt-Trucksäss et al., 2000). To further investigate these adaptations earlier post-injury, blood velocity and vessel diameter of the right CCA…"*

We hope that these adjustments adequately address the reviewer's comments and clarify the links between SCI, arterial stiffness and cognitive decline while improving the methodological clarity of our protocol manuscript.

11. There is not DSMB mentioned wish would be essential in this clinical trial.
Page 14, there is no mention of risk of using the cfPWV on anterior neck scars and metal hardware for Cx injuries.
Response: Thank you for your comment regarding the Data Safety Monitoring Board (DSMB) and the very low potential risks associated with the use of cfPWV measurements in individuals with cervical SCI with metal hardware. While we do not mention DSMB in our manuscript, we have mentioned that "*Regular executive committee meetings consisting of CI (TN), site PI (JC), site clinical lead (AO), postdoctoral research fellow (SB), research nurse, healthcare assistant and other MCSI staff will convene regularly to discuss any data collection issues or safety issues that arise*" on Page 12 of our manuscript.

Furthermore, in such cases where there is a risk of discomfort or potential irritation with the cfPWV outcome, caution is exercised to prevent any undue discomfort. We have made the following additions to the manuscript on Page 13 and 14, under Primary outcome measure: Arterial stiffness, we now state, *"The use of cfPWV as a vascular health assessment tool was carefully considered in participants with anterior neck scars or cervical SCI with implanted metal hardware. Individuals with recent surgical scars or contraindications will be excluded from cfPWV assessments to ensure their safety."*

Page 15, a citation is needed on the prognostic value of 24 hour BP monitoring, not just a methodology citation
Response: Thank you for your comment. We have added the following text to the 'Blood pressure monitoring' section, "*Furthermore, increased BP variability is a significant and independent predictor of mortality and cardiovascular events in the general population and an increased frequency of hypotensive events over 24 hours has been associated with arterial stiffening and impaired cerebrovascular reactivity in individuals with SCI.*" Additionally, we have incorporated the following references to support its prognostic value: Weber *et al.*, 2024, DOI:10.46292/sci23-00068 & Hansen *et al.*, 2010, DOI:10.1161/HYPERTENSIONAHA.109.140798.

Page 17, mention of using spare samples for other CVD health question does require permission by participants.
Response: Thank you for your comment. We completely agree that participant consent is essential when using acellular biological samples (i.e., plasma or serum) for purposes beyond the original scope of the study. Our protocol has received ethical approval for this and has outlined in our manuscript "*will be stored in accordance with the Human Tissue Act, 2004*". Furthermore, while stored samples may be used in future, our manuscript goes on to state that "*further ethical approval will be sought for additional analysis based on the General Data Protection Regulation (GDPR)*": (https://www.legislation.gov.uk/ukpga/2018/12/contents/enacted). We have also asked for consent/permission from participants to use their stored samples for other research questions, as appropriate. Please see the uploaded/attached informed consent form for the trial. We have now included a sentence in the manuscript (Page 18) to make this clear.

Page 17, I could not find the ref. on CHOICES RCT (#118).
Response: We have double checked our reference format complies with journal guidelines. However, we apologise for the inconvenience the Reviewer has had in locating this article. Hopefully one of the following identifiers will help the reviewer to locate the primary outcome manuscript from the seminal, multi-centre CHOICES exercise trial in individuals with SCI:
PMID: 30612110
PMCID: PMC6326283

DOI: 10.1136/bmjopen-2018-023540
https://bmjopen.bmj.com/content/9/1/e023540.long

VERSION 2 - REVIEW

**Reviewer**	**1**
**Name**	**Keessen, Paul**
**Affiliation**	**Amsterdam University of Applied Sciences, Faculty of Health**
**Date**	**27-Mar-2025**
**COI**	

I have no further comments.
